# Isavuconazole—Animal Data and Clinical Data

**DOI:** 10.3390/jof6040209

**Published:** 2020-10-06

**Authors:** Livio Pagano, Chiara Cattaneo, Martina Quattrone, Margherita Oberti, Maria Mazzitelli, Enrico Maria Trecarichi

**Affiliations:** 1 Dipartimento di Diagnostica per Immagini, Radioterapia Oncologica ed Ematologia, Fondazione Policlinico A. Gemelli IRCCS, 00168 Roma, Italy; 2 Sezione di Ematologia, Dipartimento di Scienze Radiologiche ed Ematologiche, Università Cattolica del Sacro Cuore, 00168 Roma, Italy; martyq211@gmail.com; 3 Divisione di Ematologia, ASST-Spedali Civili di Brescia, 25123 Brescia, Italy; chiara.cattaneo@asst-spedalicivili.it (C.C.); margherita.oberti1987@gmail.com (M.O.); 4 Department of Medical and Surgical Sciences, University of “Magna Graecia”-“Mater Domini” Teaching Hospital, 88100 Catanzaro, Italy; m.mazzitelli88@gmail.com (M.M.); em.trecarichi@unicz.it (E.M.T.)

**Keywords:** isavuconazole, antifungal prophylaxis, antifungal treatment

## Abstract

The treatment of invasive fungal infections has deeply evolved in the last years with the inclusion of new antifungals, mainly new azoles (i.e., posaconazole, isavuconazole), to the therapeutic armamentarium. This review focuses on the role of isavuconazole for treating the most important invasive fungal infections both in animals and humans (hematological and non-hematological patients).

## 1. Introduction

In the last years, new antifungal drugs have been commercialized, thus allowing an easier management of invasive fungal diseases (IFD), treatment and outcome. Isavuconazole (ISV) is a new antifungal agent, with a favorable drug-drug interaction profile, reduced drug-related adverse events and efficacy similar to voriconazole for treatment of invasive muld infections, as demonstrated in a non-inferiority trial of IFD treatment [1]. ISV demonstrated efficacy also against rare fungi and in patients with impaired renal function [2]. Due to the broad spectrum of action and to its favorable interaction profile, ISV has been proposed as a very effective antifungal drug and, mainly for patients with hematological malignancies (HMs), international guidelines strongly recommended its use for the treatment of invasive aspergillosis and mucormycosis [3,4,5].

In the present manuscript, we analyzed the literature regarding studies on animal models using ISV and the role of ISV in the treatment (target therapy and prophylaxis) of IFD in humans, stratifying the latter in non-hematological and hematological patients, in order to describe the actual clinical experiences in the use of ISV for treatment of IFDs and discuss further possible clinical applications of this drug.

## 2. Methods

A comprehensive literature search was performed using the Pubmed and Sciencedirect electronic databases. The search was performed until August 1, 2020, limiting the choice to English-language articles. All the papers that regarded “isavuconazole” were reviewed, and the relevant articles were selected. In addition, the references lists from the selected articles were used to obtain further articles not included in the electronic database. The authors reviewed all the publications identified and prepared their observations. After a revision according to the results of the plenary discussion, a summary report was made.

## 3. Isavuconazole in Animal Models

Studies conducted on animal models using ISV and focusing on dose determination, data on pharmacokinetics (PK) and pharmacodynamics (PD) parameters, and efficacy studies in animal models have demonstrated favorable results, as previously reviewed [6,7].

More recently, pharmacodynamics and efficacy of ISV have been investigated in animal models of cryptococcal meningitis. Kovanda et al. compared ISV to fluconazole and no antifungal treatment in rabbit models of cryptococcal meningoencephalitis and found similar efficacy of the two azoles in terms of reductions in the fungal burden in the brain and cerebrospinal fluid compared to untreated controls. In addition, no dose-dependent response was observed with ISV therapy, nor was a significant difference in efficacy between the two azoles observed in this study. Authors concluded suggesting that ISV could be as effective as fluconazole at least as consolidation and maintenance regimens in high-burden cryptococcal meningoencephalitis [8]. Furthermore, Wiederhold at al. compared the efficacy of treatment with oral ISV at two different dosages (120 mg/kg and 240 mg/kg BID) and fluconazole versus an untreated control group of murine models of murine cryptococcal meningitis. They found a significant improvement of survival and reductions in brain fungal burden in both treatment groups compared to controls; based on plasma and brain concentrations of ISV, they also observed better improvements in survival and fungal burden in mice treated with high-dose ISV compared to those treated with low-dose [9]. Guest et al. evaluated the efficacy of ISV in treating a murine model of *Aspergillus fumigatus* exogenous endophthalmitis. Five routes of ISV administration were performed and compared: oral gavage, intravitreal injections, intravenous injections, intravitreal injection followed by oral gavage, and intravitreal injection followed by intravenous injections. Authors observed a similar and significant improvement of the disease outcome in terms of reduction of ocular fungal burden, preservation of retinal structural integrity and function, and reduction levels of inflammatory cytokines (TNF-α, IL-1β, IL-6) and cellular infiltration in the infected eyes in all ISV administration route groups, suggesting possible beneficial effect of ISV in human ocular infections [10]. Gebremariam et al., who had previously evaluated the treatment with high dose of ISV in mice infected with *Rhizopus delemar* demonstrating that it was as effective as a high-dose liposomal amphotericin B treatment as reported in the review of Natesan et al. [7], subsequently investigated a possible beneficial effect of ISV in combination with micafungin for treatment of a murine model of pulmonary mucormycosis due to *Mucor*. In this study, authors did not find an enhanced survival of mice compared to placebo but no synergism or antagonism between the two antifungal drugs [11]. Finally, the same group assessed the efficacy of prophylaxis (treatment started on Day −2 and continued until mice infection) or continuous treatment (treatment started on Day −2 and ended on Day +2) of ISV, posaconazole and voriconazole in immunosuppressed mice with pulmonary mucormycosis due to *R. delemar*. In the prophylaxis study, an improvement in survival and fungal burden were observed only in the group of mice treated with ISV, whereas in the continuous therapy study, compared to the placebo group, improved survival and reduction of fungal burden occurred in ISV and posaconazole groups but not in voriconazole groups [12].

## 4. Isavuconazole in Hematological Patients

The efficacy of ISV in hematologic patients with IFD was first documented in the SECURE trial, a phase 3, randomized, double-blind, multicenter, non-inferiority study of ISV versus voriconazole for the primary treatment of invasive mold diseases. In this trial, more than 80% of patients were affected by HMs in both arms [1]. The study met the primary (non-inferiority of ISV versus voriconazole in the intention to treat population, ITT) and all the secondary objectives (overall response in population with proven–probable invasive mold disease, all-cause mortality at day 84, clinical/mycological/radiological response and safety tolerability). The all-cause mortality at day 42 (primary endpoint) in ITT population was 19% and 20% for ISV and voriconazole, respectively. In the ITT population with proven–probable invasive mold disease at the end of treatment (EOT), the overall response rate (ORR) was similar for ISV (35%) and voriconazole (36%), and the clinical response was 62% and 60% for ISV and voriconazole, respectively. ISV-treated patients had a lower frequency of hepatic, cutaneous and ophthalmologic adverse events. In a post hoc analysis, ISV had comparable efficacy and safety to voriconazole in neutropenic patients with invasive proven–probable aspergillosis [13]. Isavuconazole efficacy on IFD caused by rare fungi was also tested in a single-arm open-label trial (VITAL study), which enrolled patients with invasive aspergillosis and renal impairment or with rare IFD. At the EOT, the ORR in 37 (of which 59% were hematologic) patients with mucormycosis was 32%, 36% and 20% for primary treatment, for refractory disease and for intolerant to other antifungal patients, respectively, which was comparable with the response reported for liposomal amphotericin B [14]. In the same trial, a post hoc analysis showed the activity of ISV on rare fungi (both non-*Candida* yeasts and other rare molds), which confirmed its efficacy, with an overall treatment success at the EOT of 57.7% [15]. However, ISV was only marginally active on IFD caused by more than one fungal species (13.3%) [16].

Data concerning the real-life use of ISV in HMs are scanty and quite heterogeneous; moreover, the interpretation and comparison between the studies are made difficult by the different response definitions adopted. However, real-life data seem to confirm those reported by clinical trials. Ordaya et al. reported the first small series of patients treated with ISV outside clinical trials; no data about underlying disease were given [17]. Eleven out of 28 patients with probable-proven IFD received ISV because of intolerance to either posaconazole or voriconazole. The ORR was 82% and the overall mortality 18%. In a retrospective cohort study conducted on 91 adult inpatients receiving ISV for both prophylaxis and treatment for possible and proven–probable IFD, including 58 (64%) acute leukemia patients, the ORR was 42/68 (62%) in the evaluable treated patients [18]. It was higher in patients receiving ISV empirically (65%) than in salvage after another antifungal agent (53%) and no breakthrough IFD (b-IFD) were observed. To date, the largest and more specific real-life study on ISV as treatment for IFD in HMs was reported by the Sorveglianza Epidemiologica Infezioni nelle Emopatie (SEIFEM) Group [19]. In this retrospective multicenter study, data about 128 patients, all affected by HMs, including acute leukemia (AL) (67%) and allogeneic stem cell transplantation recipients (alloSCT, 33%) patients, and receiving ISV as first or second-line treatment for possible and proven–probable IFD, were reported. The ORR was 67.2% in 82/122 evaluable patients, which was similar to the clinical response observed in the SECURE trial. The ORR was similar when using ISV as a 1st- or 2nd-line treatment (60.5% vs. 70.9%, respectively, *p* = 0.24). Among patients receiving ISV as second-line treatment, those on salvage had a lower ORR (53.8%) than patients without IFD refractoriness (70.6%, *p* = 0.012). The response to ISV was similar in possible and probable IFD (68.6% and 71.2%, respectively) than in proven IFD (41.7%). Both female sex and ISV use during the induction phase of treatment for hematologic disease were predictive of a favorable ISV response. ISV tolerability was excellent in all real-life studies, with a less than 5% of permanent discontinuation. Table 1 summarizes the main results of ISV treatment in hematological malignancies patients with IFD.

Data concerning ISV distribution in central nervous system (CNS) are quite rare but seem to indicate that ISV is active also in this setting of infections. A retrospective analysis of ISV treatment in patients with CNS IFD and participating in VITAL and SECURE studies revealed a clinical response at the EOT in 21/36 patients (58.3%) and a survival rate of 69.4% (25/36) [20]. Other case reports showed the efficacy of ISV in hematologic patients affected by CNS IFD [21,22]

ISV was also employed as antifungal prophylaxis in HMs, both affected by AL and undergoing alloSCT, with less convincing results. Cornely et al. reported the results of a phase II dose escalation study designed to assess the safety and tolerability of ISV as antifungal prophylaxis in neutropenia chemotherapy-induced in acute myeloid leukemia (AML) [23]. Of 20 patients enrolled, 2 (10%) were considered as failure as they developed a possible fungal infection. A more recent open-label phase II study in AML and myelodysplastic syndrome (MDS) patients undergoing remission-induction therapy reported the development of b-IFD (2 probable—pulmonary aspergillosis—and 8 possible) in 10 (15%) of 75 enrolled patients while on ISV prophylaxis [24]. The possible explanations of these figures may be the advanced age of the population (median: 67 years) and the prolonged neutropenia, due to intensive chemotherapy or to venetoclax-based regimens. Tolerability was excellent with only marginally adverse events. ISV as antifungal prophylaxis was evaluated also in the alloSCT setting in a prospective, single-center study [25]. In this study, ISV was administered since day +7 and the maximum duration of the study was until +98. The primary end point was prophylaxis failure, defined as discontinuation for proven-probable IFD, toxicity or adverse event, need for systemic antifungal therapy for more than 14 days. Ten (10.7%) of 99 patients discontinued ISV, but only 3 (3.1%) for IFD (all candidemia); however, 4 patients had a diagnosis of possible pulmonary IFD. Seven patients discontinued ISV for toxicity, mainly hepatic. ISV as antifungal prophylaxis has been reported also in two retrospective single-center studies. Bowen et al. reported in 98 hematologic/alloSCT patients and receiving 138 courses, 14 ISV discontinuation, 6 for toxicity, and 8 for possible (2) or proven-probable (2 aspergillosis, 2 candidemia, 1 mucormycosis and 1 fusariosis) IFD [26]. Fontana et al. conducted a retrospective review of breakthrough b-IFDs in patients affected by AL or undergoing alloSCT and receiving ISV as antifungal prophylaxis for at least 7 days [27]. Proven-probable b-IFDs (1 candidemia, 7 aspergillosis, 2 fusariosis, 2 mucormycosis) were observed in 12/145 patients (8.3%), mainly acute leukemias undergoing chemotherapy. These figures were higher than historical control with posaconazole and voriconazole, and therefore, the authors decided to replace ISV with posaconazole. Two other studies on ISV prophylaxis in AL patients and alloSCT recipients are ongoing (NCT03149055, NCT03019939).

b-IFDs other than aspergillosis, including rare fungi, have been frequently reported during ISV treatment or prophylaxis. Rausch et al. evaluated 100 patients receiving antifungal prophylaxis and treatment with Isavuconazole [28]. Thirteen (13%) of patients developed a b-IFD; only one due to *Aspergillus* spp, the remaining due to *Candida* spp (6, all non-albicans species), *Trichosporon* asahii (1), *Mucorales* (4), *Fusarium* spp (1). A case of mucormycosis and 1 of scedosporidiosis beyond aspergillosis were also reported by Fung et al. in a small series of 5 b-IFDs observed in hematological patients while on ISV for prophylaxis or treatment [29].

Due to its safety profile, ISV has been immediately proposed as a winning solution in HMs affected by IFD. Beyond safety data concerning clinical trials and real-life studies [1,2,19], its tolerability has been demonstrated also in 23 acute leukemia patients discontinuing posaconazole for liver or cardiac toxicity and in 50 HMs/alloSCT patients treated for ≥6 months, without any discontinuation due to toxicity, as a confirm of the safety profile also in the “real-life” [30,31]. QTc prolongation has been considered a limiting side-effect of voriconazole and posaconazole in hematologic patients, particularly in those receiving new drugs, such as FLT3-inhibitors and venetoclax. The effect of ISV on QTc interval has been specifically explored in a multicenter study in 26 patients, including also hematologic and alloSCT patients; QTc interval was reduced in all but 2 patients, who showed no changes [32]. Occasional data of electrolyte imbalances have been reported, together with gastroenteric toxicity (nausea/vomiting) [19]. ISV excellent plasma bioavailability has been demonstrated by a post hoc analysis of the SECURE trial, where the modest variability in concentrations observed was not associated with any differences in efficacy or safety outcomes [33]. Based on these observations, ISV therapeutic drug monitoring may be considered less critical than other azoles.

ISV is a moderate inhibitor of CYP3A4 cytochrome and a mild inhibitor of P-glycoprotein efflux pump. Although its drug–drug interaction profile is modest as compared to other azoles, variability in plasma concentration of concomitant drugs which are substrates of CYP3A4 or P-glycoprotein, such as immunosuppressant (i.e., cyclosporine, tacrolimus and sirolimus) or new molecular entities (i.e., ibrutinib, venetoclax and FLT3-inhibitors), may be expected. A pharmacokinetic study in healthy subjects demonstrated an increase in plasma concentration of tacrolimus, sirolimus and cyclosporine by 125%, 84% and 29%, respectively [34]. This concentration increase has been demonstrated also in alloSCT recipients for tacrolimus and sirolimus within the first two week of administration [35]. To date, no in vivo pharmacokinetic studies have been reported in patients receiving concomitant new molecular entities. A retrospective study on patients with IFD and treated with ISV while on ibrutinib has been recently reported [36]. In this small series of 8 patients, ISV has been proved effective in 7 cases; ibrutinib was prudentially reduced in 5 cases. One patient discontinued ibrutinib for worsening of thrombocytopenia, which was considered potentially related to an increased ibrutinib exposure; both patients with progressive disease received a reduced dose of ibrutinib. These data indicate that further studies are warranted to elucidate pharmacokinetic implications of ISV and new small molecules coadministration.

## 5. Isavuconazole in Non-Hematological Patients

Clinical trials evaluating the efficacy and safety of ISV in treatment of invasive mold diseases included predominantly patients suffering from HMs, and data on patients with underlying diseases other than HMs are not specifically reported [1]. In the ACTIVE trial, a Phase 3, randomized, double-blind, non-inferiority trial comparing the efficacy and safety of intravenous (IV) ISV followed by oral ISV to IV caspofungin followed by oral voriconazole in the primary treatment of candidemia and invasive candidiasis, a total of 440 patients (400 in modified intention to treat population) were included, 221 in ISV group and 219 in caspofungin group [37]. Although patients’ underlying diseases were not specifically reported, neutropenia was present in only 25/221 (11.3%) and 24/219 (11%) patients in ISV and caspofungin group, respectively; therefore, most of the patients probably did not suffer from HMs. At primary efficacy end-point (successful overall response at the end of IV therapy) evaluation, ISV failed to demonstrate non-inferiority to caspofungin for treatment on invasive candidiasis: 60.3% of patients were successfully treated in the ISV arm and 71.1% in the caspofungin arm (adjusted difference −10.8, 95% confidence interval −19.9–−1.8). Several clinical cases have been reported regarding the use of ISV for treatment of IFD in patients suffering from clinical conditions other than HMs. After exclusion of reports published in non-English language or in which clinical and therapeutic data were not available for the purpose of the present review, a total of 41 cases have been included. Clinical and demographic characteristics, details of the antifungal treatment, outcome and adverse effects reported on these patients are summarized in Table 2 [38,39,40,41,42,43,44,45,46,47,48,49,50,51,52,53,54,55,56,57,58,59,60,61,62,63,64,65,66,67,68,69,70,71]. Thirty-three out of 42 (78.5%) patients were male and the mean age was 52 years (range 20–79). The most frequent and most important comorbidity was solid organ transplantation in a total of 6 patients (liver, *n* = 1; lung, *n* = 2; kidney, *n* = 2; heart, *n* = 1), followed by sarcoidosis (*n* = 3) and Acquired Immune Deficiency Syndrome (AIDS, *n* = 3) and Influenza A (*n* = 2). Type-2 Diabetes Mellitus was present in a total of 10 patients. In addition, the IFD was diagnosed in the context of active tuberculosis in one case and acute respiratory distress syndrome (ARDS) due to SARS-CoV-2 in another patient. The more frequent IFD treated with ISV were predominantly mucormycosis (*n* = 12), aspergillosis (*n* = 13), coccidioidal meningitis (*n* = 9) and invasive infections due to *Histoplasma capsulatum* (*n* = 3). In 7 out of 42 patients, ISV was administered as first-line therapy (often because of the presence of contraindications to other antifungals); in the remaining cases, ISV treatment was started after other first-line therapy, as a consequence of evidence of adverse effects and or clinical or microbiological failure. In some cases, ISV was started as prolonged or lifelong maintenance oral therapy. Clinical cure or stable improvement have been reported in 32 out 42 cases, whereas death or clinical and/or microbiological failure occurred in 5 cases; in two cases, treatment was discontinued due to severe adverse effects and in 3 cases the outcome was not reported. ISV treatment was generally well tolerated; in 4 cases, authors specifically stated that patients did not present adverse effects, whereas in 30 cases they were not reported. Adverse events were reported in 9/42 cases (21.4%). In six cases, gastrointestinal symptoms (*n* = 4) or hepatic injury (*n* = 2) occurred. ISV treatment was discontinued for adverse events only in 3/42 (7.1%) cases, due to nausea, vomiting, myalgia and lethargy, severe hypomagnesemia in one case, and severe liver injury and alopecia in the other two cases, respectively.

ISV has been evaluated as antifungal prophylaxis in patients diagnosed with clinical conditions other than HMs. Samanta et al. conducted a single-center, retrospective study comparing efficacy and safety of ISV (144 patients) versus voriconazole (156 patients) as antifungal prophylaxis in lung transplant recipients; of note, adjunctive inhaled amphotericin B was also administered to 100% and 41% of patients in ISV and voriconazole groups, respectively [72]. At 1-year follow-up, no difference in IFD occurrence was reported between the two groups (7% vs. 8%). Authors also identified red blood cell transfusion >7 units at transplant mold-positive respiratory culture as independent risk factors for both breakthrough IFD and breakthrough invasive mold infections, whereas the African-American race was independently associated specifically with breakthrough IFD and bronchial necrosis >2 cm from anastomosis and basiliximab induction were independent risk factors only for invasive mold infections. ISV was tolerated significantly better than voriconazole (premature discontinuation rates due to adverse events: 11% vs. 36% of patients, respectively). Finally, antifungal prophylaxis prolonged for ≥90 days was associated with a significantly lower rate of IFD at 1-year follow-up (3% vs. 9%). ISV for prophylaxis in a patient undergoing lung transplantation was used also in another patient who had developed QTc prolongation while receiving voriconazole; ISV was well tolerated with normalization of QTc level and no subsequent occurrence of IFD was reported [73]. Finally, ISV was used as occupational post-exposure prophylaxis after a deep cut with a scalpel used for processing a clinical sample contaminated by *Rhizopus* spp.; prophylaxis was discontinued after two weeks because of side effects (severe nausea and diarrhea), and no subsequent local fungal infection was reported [74].

## 6. Discussion

### 6.1. Proper Summary

Registrative trials demonstrated that ISV has a similar efficacy to voriconazole for the treatment of invasive aspergillosis and to liposomal amphotericin B for the treatment of mucormycosis [1,16]. Efficacy against rare fungi has also been reported [15]. On the contrary, ISV did not prove its efficacy on candidemia and invasive candidiasis as it failed to demonstrate the non-inferiority to caspofungin [38].

The role of ISV in target therapy is well defined both in HMs and non-HMs patients, and data from real life confirm the efficacy of this antifungal agent. Animal models and sporadic reports in humans seem to indicate its efficacy also in CNS infections.

ISV has been reported better tolerated than voriconazole in the SECURE study [1], and real life data confirm this safety profile [18,19]. Considering the lower side effect incidence and reduced drug–drug interactions, a possible application in prophylaxis, use not licensed at present, has been evaluated, although b-IFDs, including rare fungi, have been frequently reported and this phenomenon needs close surveillance.

### 6.2. Conclusions

Isavuconazole, in conclusion, has proven to be effective as a treatment both against aspergillosis and against other molds, with the advantage of better handling respect of the other azoles. Accordingly, international guidelines indicated ISV as a useful alternative to voriconazole (i.e., the current gold standard) and the other available agents in the treatment of invasive aspergillosis mainly in patients with HMs [3,4]. In addition, the recent Global Guidelines for Mucormycosis indicated ISV, with liposomal amphotericin B, for the treatment of mucormycosis [5].

### 6.3. Future Perspectives

Although non-registration clinical studies do not attest convincing results in prophylaxis at present, further studies are warranted to explore this type of approach, which would be very attractive in patients receiving as concomitant treatment drugs metabolized via CYP450 and P-glycoprotein efflux pump pathway, given the modest inhibition of the CYP450 system by ISV. Moreover, the efficacy of ISV in treatment of CNS IFDs seems very promising and should be verified, as the choice of ISV in this setting may also offer the possibility of very prolonged treatment without significant toxicities. Another possible future application of ISV could be in combination with other antifungal drugs for the treatment of very aggressive and refractory IFDs or for mixed IFDs; however, at present, there is no evidence that this approach could be a benefit for the treatment of this peculiar subset of IFDs.

## Figures and Tables

**Table:** **Table 1.** Isavuconazole treatment in hematological malignancies patients with IFD.

Author, Year (Ref)	Type of Study	Patient’s Number (% Hematologic Patients)	ORR	Clinical Response§	Notes
Maertens et al., 2016 [1]	Randomized, double-blind	258 (82%)	50/143 (35%)	85/137 * (62%)	-Proven/probable IFD 143/258 (55%) -Aspergillosis 84%
Marty et al., 2016 [2]	Single-arm, open label	37 (59%)	11/35 * (31%)	14/31 * (45%)	-Proven/probable mucormycosis
Cornely et al., 2018 [15]	Single-arm, open label	26 (54%)	NR	15/26 (57.7%)	-Proven/probable mold (24) or non-*Candida* yeast (2) infections
Hassouna et al., 2019 [18]	Retrospective, single-center	91 ** (69%)	42/68 * (62%)	NR	-Proven/probable (45%) and possible (55%) IFD -17/38 aspergillosis and 10/38 mucormycosis
Cattaneo et al., 2019 [19]	Retrospective, multicenter	128 (100%)	80/122 * (65.5%)	82/122 * (67.2%)	-Proven/probable (58%) and possible (42%) IFD -66/71 (93%) aspergillosis

ORR: overall response rate (complete+partial response); § Clinical response: complete or partial resolution of all, some or none of the attributable clinical symptoms and physical findings; * evaluable patients; ** 85 received isavuconazole as treatment, 6 as prophylaxis.

**Table 2:** Isavuconazole treatment in non-hematological patients with invasive fungal infections.

Author, Year (ref)	No. of Patients	Age, Gender	Comorbidities	Invasive Fungal Infection	Previous Antifungal Therapy	Reason for Isavuconazole Treatment	Duration of Isavuconazole Therapy	Combination Therapy	Outcome	Adverse Effects
Ervens J. et al., 2014 [38]	1	45, M	Ulcerative colitis Hypertension, Previous unilateral renal loss	Rhinocerebral mucormycosis (*Rhizopus oryzae*)	Liposomal Amphotericin B plus Posaconazole	LAmB, acute renal failure and microbiological failure POS, insufficient therapeutic plasma level and microbiological failure	506 days	None	Recovery	NR
Knoll B. et al., 2014 [39]	1	47, M	Previous laparoscopic Rouxen-Y gastric bypass surgery in 2007 Diabetes mellitus	Lung angioinvasive mucormycosis (*Rhizopus* spp.)	Liposomal Amphotericin B plus Posaconazole	LAmB, acute renal POS, insufficient therapeutic plasma level	4 months	None	Recovery	Mild nausea (treatment not discontinued)
Ahmed Y. et al., 2016 [40]	1	53, M	AIDS Diabetes mellitus	Acute invasive fungal rhinosinusitis (*Rhizopus* spp.)	Liposomal Amphotericin B plus micafungin	LAmB, acute renal failure and failure MIC, failure	Still on ISV (22 days at least)	None	Clinical improvement	Mild liver injury (treatment not discontinued)
Morales M.K. et al., 2016 [72]	1	48, F	Liver transplantation Asthma	Pulmonary aspergillosis	Voriconazole Liposomal Amphotericin B	VOR, angioedema LAmB, NR	Ongoing	None	NR	None
Jacobs SE et al., 2017 [42]	1	56, F	Asthma Corticosteroid therapy	Allergic bronco-polmunary aspergillosis	Itraconazole Voriconazole	ITR, hives VOR, hepatic and neurological toxicity	10 weeks	None	Relapse after few months, with a new course of ISV for 5 months and subsequent clinical improvement	Watery diarrhea (treatment not discontinued)
Wiley Z et al., 2017 [43]	1	56, M	None	*Histoplasma capsulatum* endocarditis	Liposomal Amphotericin B Itraconazole	LAmB, acute renal failure ITR, drug interactions and QT prolongation	Long-life	None	Clinical improvement	NR
Bansal R et al., 2017 [44]	1	57, M	Sarcoidosis Alcohol abuse	Disseminated mucormicosis (gastric and bladder)	Amphotericin B (systemic and via bladder irrigation)	Switch to oral maintenance therapy	6 months	None	NR	NR
Bongomin F et al., 2018 [45]	1	55, M	Sarcoidosis Previously treated pulmonary tuberculosis Early mild cirrhosis	Chronic pulmonary aspergillosis (*A. fumigatus*)	Itraconazole, Voriconazole	ITR, breakthrough in vitro resistance VOR, insufficient therapeutic plasma level	11 months	None	Clinical improvement	Liver injury (treatment discontinuation)
Burston J et al., 2018 [46]	1	73, M	Diabetes Mellitus Ischemic heart disease Hypertension	Rhinoorbital mucormycosis (*Rhizopus arrhizus*)	Liposomal Amphtericin B, Posaconazole	LAmB, acute renal failure POS, severe symptomatic hypomagnesemia	17 days	None	Discontinuation for adverse effects	Nausea, vomiting, myalgia and lethargy, severe hypomagnesemia (treatment discontinuation)
Shafiq M et al., 2018 [47]	1	67, M	Type-2 Diabetes Mellitus Hypertension,	Rhinoorbital mucormycosis complicated by meningitis	Liposomal Amphtericin B	LAmB, Failure	4 months	Liposomal Amphotericinn B (50 days)	Recovery	Headaches (treatment not discontinued)
Arsiè E et al., 2018 [48]	1	58, M	Decompensate cirrhosis	Pulmonary aspergillosis	Liposomal Amphotericin B	LAmB, acute infusion-related reaction to the first infusion	NR	None	Death for multiorgan failure	NR
Kabulski GM et al., 2018 [49]	1	20, F	Lung transplantation Cystic fibrosis Sinusitis Pancreatic insufficiency	Pulmonary mucormycosis	None	-	6 months	None.	Recovery	Nausea, diarrhea abdominal pain (treatment not discontinued)
Huggins J et al., 2018 [50]	1	62, M	Sarcoidosis, Non-alcoholic steatohepatitis-related cirrhosis Type-II Diabetes Mellitus	Polmunary mucormycosis (*Rhizopus oryzae*)	Liposomal Amphotericin B	LAmB, acute renal failure	6 weeks	None	Recovery	None
Bassetti M. et al., 2018 [51]	1	69, M	COPD Suspected X-linked granulomatous disease	Probable Invasive pulmonary aspergillosis	None	-	Three months	None	Recovery	None
Castro-Lainez M.T. et al., 2018 [52]	1	75, M	Bullous emphysema	Pneumonia due to *Talaromyces (Penicillium) marneffei*.	Voriconazole	VOR, side effects	28 days	Liposomal Amphotericin B	Recurrence and subsequent death	NR
Jariwal R. et al., 2018 [53]	1	35, F	Previous rhino-cerebral aspergillosis treated surgically and with voriconazole	Recurrence of rhino-cerebral aspergillosis	None	-	Long-life	None	Recovery	NR
Thielen BK et al., 2018 [54]	1	63, M	Full-thickness burns of approximately 47% of total body surface Type-2 diabetes mellitus Ischemic cardiomyopathy	Mucormycosis due to *Lichtheimia* spp.	Posaconazole	POS, failure	Dose not reported 6 weeks	Topical amphotericin B washes.	Recovery	NR
Linder KA et al., 2019 [55]	1	60, M	ARDS due to Influenza A Type-2 diabetes mellitus Factor V Leiden heterozygosity	*Cryptococcus neoformans* pulmonary infection	Fluconazole	FLU, QT prolongation	6 days	None	Clinical and microbiological failure	NR
Adamsick ML et al., 2019 [56]	1	68, M	Lung transplantation End-stage renal disease on haemodialysis	Probable invasive aspergillosis	None	-	NR	None	NR	NR
Ilharco M et al., 2019 [57]	1	61, M	Chronic sinusitis Dyslipidemia	Rhinoorbital mucormycosis	Liposomal Amphotericin B plus Posaconazole	Failure	24 days intravenously, more than seven months orally	None	Recovery	NR
Mazzella A et al., 2020 [58]	1	30, M	AIDS	Disseminated histoplasmosis	Liposomal Amphotericin B (backbone therapy) Itraconazole Posaconazole	ITR, failure for insufficient therapeutic plasma level POS, insufficient therapeutic plasma level	One year	Liposomal Amphotericin B, until clinical improvement	Recovery	NR
Guillen-Vera D et al., 2019 [41]	1	77, F	Nonatopic severe persistent asthma Prolonged corticosteroid therapy Ovarian cancer	Chronic pulmonary aspergillosis	Voriconazole Posaconazole	VOR, neurological toxicity POS, possible drug interactions	4 months	None	Recovery	NR
Gani I et al., 2019 [59]	1	79, M	Renal transplantation Hypertension Type-2 diabetes mellitus Coronary artery disease	Gastric mucormycosis due to *Rhizopus* spp.	None	-	Life-long	None	Recovery	NR
Buonomo AR et al., 2019 [60]	1	48, M	Crohn’s disease treated with infliximab Active pulmonary tuberculosis	Probable pulmonary aspergillosis	Liposomal Amphotericin B	Switch to oral maintenance therapy	16 weeks	No	Recovery	NR
Canfield GS et al., 2019 [61]	1	55, M	Renal transplantation	Posttransplant Immune Reconstitution Syndrome in *Cryptococcus gattii* meningo-encephalitis	Liposomal Amphotericin B plus flucytosine as induction therapy Fluconazole as maintenance therapy,	FLU, QTc prolongation	NR	No	Recovery	NR
Finnis M et al., 2020 [62]	1	52, F	AIDS Coronary artery disease, Hypertension, Cerebral vascular accident, COPD	Gastrointestinal histoplasmosis with esophageal involvement	Itraconazole	ITR, patient’s intolerance	NR	No	Clinical improvement	NR
Koehler P et al., 2020 [63]	1	70, M	ARDS due to COVID-19	Pulmonary aspergillosis	None	-	NR	No	Death	NR
Assaf A et al., 2020 [64]	1	65, M	Heart transplantation	*Aspergillus fumigatus* sternal osteomyelitis	Voriconazole Liposomal Amphotericin B	VOR, neurologic and cutaneous toxicities LAmB, renal toxicity	10 months	No	Clinical cure	NR
Prabhudas-Strycker KK et al., 2020 [65]	1	41, M	End stage renal disease on hemodialysis Hypertension	*Candida tropicalis* infective endocarditis.	Amphotericin B Micafungin (induction therapy)	AmB, patient’s intolerance (back pain)	Long-life suppression therapy	No	No recurrence at 1-year follow-up	NR
Abreu I et al., 2020 [66]	1	24, M	Elective pleurodesis for recurrent spontaneous pneumothorax	Pleural aspergillosis	Voriconazole Liposomal Amphotericin B	VOR, hepatotoxicity LAmB, renal toxicity	65 days	No	Recovery	None
Hoang K et al., 2020 [67]	1	66, M	Influenza A Type-2 Diabetes Mellitus Corticosteroid therapy	Pulmonary mucormycosis due to *Rhizopus* spp.	None	-	21 days	No	Clinical and radiological failure	NR
Heidari A et al., 2019 [68]	9	46, M	None	Coccidioidal meningitis	Fluconazole Posaconazole, Voriconazole	FLU, failure; POS, gastrointestinal toxicity; VOR neurological toxicity	621 days	No	Recovery	NR
		49, F	None	Coccidioidal meningitis	Fluconazole Voriconazole	VOR, failure	441 days	No	Recovery	NR
		44, M	None	Coccidioidal meningitis	Fluconazole	FLU, failure	518 days	No	Recovery	NR
		33, M	None	Coccidioidal meningitis	Fluconazole Voriconazole	VOR, cutaneous toxicity	637 days	No	Clinical stability	NR
		55, M	None	Coccidioidal meningitis	Fluconazole Voriconazole	VOR, gastrointestinal toxicity	138 days	No	Clinical stability	NR
		51, M	None	Coccidioidal meningitis	Fluconazole Voriconazole	VOR, cutaneous toxicity	708 days	No	Clinical stability	NR
		41, M	None	Coccidioidal meningitis	Fluconazole Voriconazole	VOR, cutaneous toxicity	274 days	No	Clinical stability	NR
		43, M	None	Coccidioidal meningitis	Fluconazole Voriconazole	VOR, cutaneous toxicity	385 days	No	Clinical stability	NR
		59, M	None	Coccidioidal meningitis	Fluconazole Voriconazole Fluconazole	FLU, failure; VOR, hepatic injury; FLU, gastrointestinal toxicity	810 days	No	Clinical stability	NR
Kiley JL et al., 2019 [69]	1	24, F	Migraine	Toxic epidermal necrolysis By *Thricosporon asahii*	None		23 days	No	Recovery	NR
Routray C et al., 2020 [70]	1	65, F	Type-2 Diabetes Mellitus Three vessel coronary artery disease	Sternal osteomyelitis secondary to *Aspergillus fumigatus* after cardiothoracic surgery	Micafungin Voriconazole	VOR, intractable nausea and poor appetite	NR	No	Discontinuation for adverse effects	Alopecia

NR, not reported; NA, not available; AmB, Amphotericin B; LAmB, liposomal Amphotericin B; VOR, voriconazole; ITR, itravuconazole; POS, posaconazole; MIC, micafungin; COPD, chronic obstructive pulmonary disease; AIDS, acquired immunodeficiency syndrome; ARDS, acute respiratory distress syndrome; COVID-19, coronavirus diseases 19; “-“, not applicable.

## References

[1] Maertens J.A., Raad I.I., Marr K.A., Patterson T.F., Kontoyiannis D.P., Cornely O.A., Bow E.J., Rahav G., Neofytos D., Aoun M. (2016). Isavuconazole versus voriconazole for primary treatment of invasive mould disease caused by Aspergillus and other filamentous fungi (SECURE): A phase 3, randomised-controlled, non-inferiority trial. Lancet.

[2] Marty F.M., Ostrosky-Zeichner L., Cornely O.A., Mullane K.M., Perfect J.R., Thompson G.R., Alangaden G.J., Brown J.M., Fredricks D.N., Heinz W.J. (2016). Isavuconazole treatment for mucormycosis: A single-arm open-label trial and case-control analysis. Lancet Infect. Dis..

[3] Tissot F., Agrawal S., Pagano L., Petrikkos G., Groll A.H., Skiada A., Lass-Flörl C., Calandra T., Viscoli C., Herbrecht R. (2017). ECIL-6 guidelines for the treatment of invasive candidiasis, aspergillosis and mucormycosis in leukemia and hematopoietic stem cell transplant patients. Haematologica.

[4] Ullmann A.J., Aguado J.M., Arikan-Akdagli S., Denning D.W., Groll A.H., Lagrou K., Lass-Flörl C., Lewis R.E., Munoz P., Verweij P.E. (2018). Diagnosis and management of Aspergillus diseases: Executive summary of the 2017 ESCMID-ECMM-ERS guideline. Clin. Microbiol. Infect..

[5] Cornely O., Alastruey-Izquierdo A., Arenz D., Chen S., Dannaoui E., Hochhegger B., Hoenigl M., Jensen H., Lagrou K., Lewis R. (2019). Global guideline for the diagnosis and management of mucormycosis: An initiative of the European Confederation of Medical Mycology in cooperation with the Mycoses Study Group Education and Research Consortium. Lancet Infect Dis..

[6] McCarthy M.W., Moriyama B., Petraitiene R., Walsh T.J., Petraitis V. (2018). Clinical Pharmacokinetics and Pharmacodynamics of Isavuconazole. Clin. Pharmacokinet..

[7] Natesan S.K., Chandrasekar P.H. (2016). Isavuconazole for the treatment of invasive aspergillosis and mucormycosis: Current evidence, safety, efficacy, and clinical recommendations. Infect. Drug Resist..

[8] Kovanda L.L., Giamberardino C., McEntee L., Toffaletti D.L., Franke K.S., Bartuska A., Smilnak G., De Castro G.C., Ripple K., Hope W.W. (2019). Pharmacodynamics of isavuconazole in a rabbit model of cryptococcal meningoencephalitis. Antimicrob. Agents Chemother..

[9] Wiederhold N.P., Kovanda L., Najvar L.K., Bocanegra R., Olivo M., Kirkpatrick W.R., Patterson T.F. (2016). Isavuconazole is effective for the treatment of experimental cryptococcal meningitis. Antimicrob. Agents Chemother..

[10] Guest J.M., Singh P.K., Revankar S.G., Chandrasekar P.H., Kumar A. (2018). Isavuconazole for Treatment of Experimental Fungal Endophthalmitis Caused by Aspergillus fumigatus. Antimicrob. Agents Chemother..

[11] Gebremariam T., Wiederhold N.P., Alqarihi A., Uppuluri P., Azie N., Edwards J.E., Ibrahim A.S. (2016). Monotherapy or combination therapy of isavuconazole and micafungin for treating murine mucormycosis. J. Antimicrob. Chemother..

[12] Gebremariam T., Alkhazraji S., Baldin C., Kovanda L., Wiederhold N.P., Ibrahim A.S. (2017). Prophylaxis with isavuconazole or posaconazole protects immunosuppressed mice from pulmonary mucormycosis. Antimicrob. Agents Chemother..

[13] Kontoyiannis D.P., Selleslag D., Mullane K., Cornely O.A., Hope W., Lortholary O., Croos-Dabrera R., Lademacher C., Engelhardt M., Patterson T.F. (2018). Impact of unresolved neutropenia in patients with neutropenia and invasive aspergillosis: A post hoc analysis of the SECURE trial. J. Antimicrob. Chemother..

[14] Kara I.O., Tasova Y., Uguz A., Sahin B. (2009). Mucormycosis-associated fungal infections in patients with haematologic malignancies. Int. J. Clin. Pract..

[15] Cornely O.A., Mullane K.M., Ostrosky-Zeichner L., Maher R.M., Croos-Dabrera R., Lu Q., Lademacher C., Perfect J.R., Oren I., Schmitt-Hoffmann A.H. (2018). Isavuconazole for treatment of rare invasive fungal diseases. Mycoses.

[16] Marty F.M., Cornely O.A., Mullane K.M., Ostrosky-Zeichner L., Maher R.M., Croos-Dabrera R., Lu Q., Lademacher C., Oren I., Schmitt-Hoffmann A.H. (2018). Isavuconazole for treatment of invasive fungal diseases caused by more than one fungal species. Mycoses.

[17] Ordaya E.E., Alangaden G.J. (2016). Real-life use of isavuconazole in patients intolerant to other azoles. Clin. Infect. Dis..

[18] Hassouna H., Athans V., Brizendine K.D. (2019). Real-world use—Isavuconazole at a large academic medical center. Mycoses.

[19] Cattaneo C., Busca A., Gramegna D., Farina F., Candoni A., Piedimonte M., Fracchiolla N., Pagani C., Del Principe M.I., Tisi M.C. (2019). Isavuconazole in Hematological Patients: Results of a Real-Life Multicentre Observational Seifem Study. HemaSphere.

[20] Schwartz S., Cornely O.A., Hamed K., Marty F.M., Maertens J., Rahav G., Herbrecht R., Heinz W.J. (2020). Isavuconazole for the treatment of patients with invasive fungal diseases involving the central nervous system. Med. Mycol..

[21] Rouzaud C., Jullien V., Herbrecht A., Palmier B., Lapusan S., Morgand M., Guéry R., Dureault A., Danion F., Puget S. (2019). Isavuconazole Diffusion in Infected Human Brain. Antimicrob. Agents. Chemother..

[22] Perrone S., Lisi C., La Barbera E.O., Luise C., Lichtner M., Girmenia C., Cimino G. (2020). Isavuconazole Therapy of Disseminated and Encephalic *Saprochaete Capitata* Infection in an Acute Myeloid Leukemia Patient Treated with Midostaurin. Mediterr. J. Hematol. Infect. Dis..

[23] Cornely O.A., Böhme A., Schmitt-Hoffmann A., Ullmann A.J. (2015). Safety and pharmacokinetics of isavuconazole as antifungal prophylaxis in acute myeloid leukemia patients with neutropenia: Results of a phase 2, dose escalation study. Antimicrob. Agents Chemother..

[24] Bose P., McCue D., Wiederhold N.P., Kadia T.M., Borthakur G., Ravandi F., Yilmaz M., Takahashi K., Thompson P.A., Pemmaraju N. (2020). Isavuconazole (ISAV) As Primary Anti-Fungal Prophylaxis in Acute Myeloid Leukemia or Myelodysplastic Syndrome: An Open-Label, Prospective Study. Clin Infect Dis..

[25] Stern A., Su Y., Lee Y.J., Seo S., Shaffer B., Tamari R., Gyurkocza B., Barker J., Bogler Y., Giralt S. (2020). A Single-Center, Open-Label Trial of Isavuconazole Prophylaxis against Invasive Fungal Infection in Patients Undergoing Allogeneic Hematopoietic Cell Transplantation: Isavuconazole for Antifungal Prophylaxis following HCT. Biol. Blood Marrow Transplant..

[26] Bowen C.D., Tallman G.B., Hakki M., Lewis J.S. (2019). Isavuconazole to prevent invasive fungal infection in immunocompromised adults: Initial experience at an academic medical centre. Mycoses.

[27] Fontana L., Perlin D.S., Zhao Y., Noble B.N., Lewis J.S., Strasfeld L., Hakki M. (2020). Isavuconazole prophylaxis in patients with hematologic malignancies and hematopoietic cell transplant recipients. Clin. Infect. Dis..

[28] Rausch C.R., Dipippo A.J., Bose P., Kontoyiannis D.P. (2018). Breakthrough fungal infections in patients with leukemia receiving isavuconazole. Clin. Infect. Dis..

[29] Fung M., Schwartz B.S., Doernberg S.B., Langelier C., Lo M., Graff L., Tan M., Logan A.C., Chin-Hong P., Babik J.M. (2018). Breakthrough Invasive Fungal Infections on Isavuconazole Prophylaxis and Treatment: What Is Happening in the Real-World Setting. Clin. Infect. Dis..

[30] DiPippo A.J., Rausch C.R., Kontoyiannis D.P. (2019). Tolerability of isavuconazole after posaconazole toxicity in leukaemia patients. Mycoses.

[31] Dipippo A.J., Kontoyiannis D.P. (2019). Lack of Toxicity with Long-term Isavuconazole Use in Patients with Hematologic Malignancy. Clin. Infect. Dis..

[32] Mellinghoff S.C., Bassetti M., Dörfel D., Hagel S., Lehners N., Plis A., Schalk E., Vena A., Cornely O.A. (2018). Isavuconazole shortens the QTc interval. Mycoses.

[33] Kaindl T., Andes D., Engelhardt M., Saulay M., Larger P., Groll A.H. (2019). Variability and exposure-response relationships of isavuconazole plasma concentrations in the Phase 3 SECURE trial of patients with invasive mould diseases. J. Antimicrob. Chemother..

[34] Groll A.H., Desai A., Han D., Howieson C., Kato K., Akhtar S., Kowalski D., Lademacher C., Lewis W., Pearlman H. (2017). Pharmacokinetic Assessment of Drug-Drug Interactions of Isavuconazole With the Immunosuppressants Cyclosporine, Mycophenolic Acid, Prednisolone, Sirolimus, and Tacrolimus in Healthy Adults. Clin. Pharmacol. Drug Dev..

[35] Kieu V., Jhangiani K., Dadwal S., Nakamura R., Pon D. (2019). Effect of isavuconazole on tacrolimus and sirolimus serum concentrations in allogeneic hematopoietic stem cell transplant patients: A drug-drug interaction study. Transpl. Infect. Dis..

[36] Cummins K.C., Cheng M.P., Kubiak D.W., Davids M.S., Marty F.M., Issa N.C. (2019). Isavuconazole for the treatment of invasive fungal disease in patients receiving ibrutinib. Leuk. Lymphoma.

[37] Kullberg B.J., Viscoli C., Pappas P.G., Vazquez J., Ostrosky-Zeichner L., Rotstein C., Sobel J.D., Herbrecht R., Rahav G., Jaruratanasirikul S. (2019). Isavuconazole Versus Caspofungin in the Treatment of Candidemia and Other Invasive Candida Infections: The ACTIVE Trial. Clin. Infect. Dis..

[38] Ervens J., Ghannoum M., Graf B., Schwartz S. (2014). Successful isavuconazole salvage therapy in a patient with invasive mucormycosis. Infection.

[39] Knoll B.M. (2014). Pharmacokinetics of oral isavuconazole in a patient after Roux-en-Y gastric bypass surgery. J. Antimicrob. Chemother..

[40] Ahmed Y., Delaney S., Markarian A. (2016). Successful Isavuconazole therapy in a patient with acute invasive fungal rhinosinusitis and acquired immune deficiency syndrome. Am. J. Otolaryngol..

[41] Guillen-Vera D., Ruiz-Ruigómez M., García-Moguel I., Morales-Ruiz R., Corbella L., Fernández-Rodríguez C. (2019). Successful treatment of chronic pulmonary aspergillosis with isavuconazole. J. Investig. Allergol. Clin. Immunol..

[42] Jacobs S.E., Saez-Lacy D., Wynkoop W., Walsh T.J. (2017). Successful treatment of allergic bronchopulmonary aspergillosis with isavuconazole: Case report and review of the literature. Open Forum Infect. Dis..

[43] Wiley Z., Woodworth M.H., Jacob J.T., Lockhart S.R., Rouphael N.G., Gullett J.C., Guarner J., Workowski K. (2017). Diagnostic Importance of Hyphae on Heart Valve Tissue in Histoplasma Endocarditis and Treatment With Isavuconazole. Open Forum Infect. Dis..

[44] Bansal R., Duddempudi S., Thelmo W., Rajnish L. (2017). A unique case of upper GI bleed. Acta Gastroenterol. Belg..

[45] Bongomin F., Rodriguez-Goncer I., Lorden C., Otu A., Bazaz R. (2018). Late-onset isavuconazole-induced liver injury. Med. Mycol. Case Rep..

[46] Burston J., Robertson M., van Hal S., Pinto A.N. (2018). Posaconazole and isavuconazole induced hypomagnesaemia. Med. Mycol. Case Rep..

[47] Shafiq M., Ali Z., Ukani R., Brewer J. (2018). Isavuconazole: A Promising Salvage Therapy for Invasive Mucormycosis. Cureus.

[48] Arsiè E., Piconi S., Iavarone M., Cozzi V., Lampertico P., Cattaneo D. (2018). High isavuconazole plasma levels in a patient with possible invasive pulmonary aspergillosis and cirrhosis. Eur. J. Clin. Pharmacol..

[49] Kabulski G.M., MacVane S.H. (2018). Isavuconazole pharmacokinetics in a patient with cystic fibrosis following bilateral orthotopic lung transplantation. Transpl. Infect. Dis..

[50] Huggins J., Al Jurdi A., Gupta R. (2018). Breaking the mold: A case of pulmonary mucormycosis treated with isavuconazole. Med. Mycol. Case Rep..

[51] Bassetti M., Carnelutti A., Righi E. (2018). Issues in the management of invasive pulmonary aspergillosis in non-neutropenic patients in the intensive care unit: A role for isavuconazole. IDCases.

[52] Castro-Lainez M.T., Sierra-Hoffman M., LLompart-Zeno J., Adams R., Howell A., Hoffman-Roberts H., Fader R., Arroliga A.C., Jinadatha C. (2018). Talaromyces marneffei infection in a non-HIV non-endemic population. IDCases.

[53] Jariwal R., Heidari A., Sandhu A., Patel J., Shoaepour K., Natarajan P., Cobos E. (2018). Granulomatous Invasive Aspergillus flavus Infection Involving the Nasal Sinuses and Brain. J. Investig. Med. High Impact Case Reports.

[54] Thielen B.K., Barnes A.M.T., Sabin A.P., Huebner B., Nelson S., Wesenberg E., Hansen G.T. (2019). Widespread Lichtheimia Infection in a Patient with Extensive Burns: Opportunities for Novel Antifungal Agents. Mycopathologia.

[55] Linder K.A., Gandhi T.N., Miceli M.H. (2019). Treatment Failure of Isavuconazole in a Patient with Cryptococcosis. Mycopathologia.

[56] Adamsick M.L., Elshaboury R.H., Gift T., Mansour M.K., Kotton C.N., Gandhi R.G. (2019). Therapeutic drug concentrations of isavuconazole following the administration of isavuconazonium sulfate capsules via gastro-jejunum tube: A case report. Transpl. Infect. Dis..

[57] Ilharco M., Pereira C.M., Moreira L., Proença A.L., do Carmo Fevereiro M., Lampreia F., Oliveira M.L., Rola J. (2019). Rhinoorbital mucormycosis in the immunocompetent: Experience with Isavuconazole. IDCases.

[58] Mazzella A., Stone N.R.H., Pool E.R.M., García Mingo A., Bolache S., Wood C. (2020). HIV-associated disseminated histoplasmosis successfully treated with isavuconazole consolidation therapy. Med. Mycol. Case Rep..

[59] Gani I., Doroodchi A., Falkenstrom K., Berry H., Lee W., Mulloy L., Saeed M., Kapoor R. (2019). Gastric Mucormycosis in a Renal Transplant Patient Treated with Isavuconazole Monotherapy. Case Rep. Transplant..

[60] Buonomo A.R., Viceconte G., Compare D., Vargas M., Iacovazzo C., Zappulo E., Nardone G., Servillo G., Borgia G., Gentile I. (2019). Invasive pulmonary aspergillosis and pulmonary tuberculosis in a patient treated with infliximab for Crohn’s disease. IDCases.

[61] Canfield G.S., Henao-Martínez A.F., Franco-Paredes C., Zhelnin K., Wilson M.L., Shihadeh K.C., Wyles D., Gardner E.M. (2019). Corticosteroids for Posttransplant Immune Reconstitution Syndrome in Cryptococcus gattii Meningoencephalitis: Case Report and Literature Review. Open Forum Infect. Dis..

[62] Finniss M., Lewis P., Myers J., Ibrahim L., Patel P. (2020). A case of gastrointestinal histoplasmosis with esophageal involvement. Clin. J. Gastroenterol..

[63] Koehler P., Cornely O.A., Böttiger B.W., Dusse F., Eichenauer D.A., Fuchs F., Hallek M., Jung N., Klein F., Persigehl T. (2020). COVID-19 associated pulmonary aspergillosis. Mycoses.

[64] Assaf A., Faure E., Sermet K., Loridant S., Leroy J., Goeminne C., Dozier A., Chopin M.C., Panaget S., Faure K. (2020). Successful treatment of Aspergillus fumigatus sternal osteomyelitis with isavuconazole in a heart transplant recipient. Transpl. Infect. Dis..

[65] Prabhudas-Strycker K.K., Butt S., Reddy M.T. (2020). Candida tropicalis endocarditis successfully treated with AngioVac and micafungin followed by long-term isavuconazole suppression. IDCases.

[66] Abreu I., Guedes M., Duro R., Lopes S., Maciel J., Santos L. (2020). Pleural aspergillosis in a patient with recurrent spontaneous pneumothorax: The challenge of an optimal therapeutic approach. Med. Mycol. Case Rep..

[67] Hoang K., Abdo T., Reinersman J.M., Lu R., Higuita N.I.A. (2020). A case of invasive pulmonary mucormycosis resulting from short courses of corticosteroids in a well-controlled diabetic patient. Med. Mycol. Case Rep..

[68] Heidari A., Quinlan M., Benjamin D.J., Laurence B., Mu A., Ngai T., Hoffman W.J., Cohen S.H., McHardy I., Johnson R. (2019). Isavuconazole in the treatment of coccidioidal meningitis. Antimicrob. Agents Chemother..

[69] Kiley J.L., Zack J., Ritchie S., Krauland K., Markelz E. (2019). Deep cutaneous Trichosporon asahii infection in a patient recovering from toxic epidermal necrolysis. Med Mycol Case Reports..

[70] Routray C., Nwaigwe C. (2020). Sternal osteomyelitis secondary to Aspergillus fumigatus after cardiothoracic surgery. Med. Mycol Case Rep..

[71] Morales M.K., Harris C., Shoham S. (2017). Graded isavuconazole introduction in a patient with voriconazole allergy. Transpl Infect Dis..

[72] Samanta P., Clancy C.J., Marini R.V., Rivosecchi R.M., McCreary E.K., Shields R.K., Falcione B.A., Viehman A., Sacha L., Kwak E.J. (2020). Isavuconazole is as effective as and better tolerated than voriconazole for antifungal prophylaxis in lung transplant recipients. Clin. Infect. Dis..

[73] Trang T.P., Hanretty A.M., Langelier C., Yang K. (2017). Use of isavuconazole in a patient with voriconazole-induced QTc prolongation. Transpl. Infect. Dis..

[74] Al-Obaidi M., Younes P., Ostrosky-Zeichner L. (2018). Post-exposure prophylaxis with isavuconazole after occupational exposure to Rhizopus. Oxford Med. Case Reports.

